# Impact of the COVID-19 Pandemic on Diabetic Ketoacidosis Patients Treated in a Pediatric Intensive Care Unit: A Single-Center Cross-Sectional Study

**DOI:** 10.3390/medicina60111775

**Published:** 2024-10-30

**Authors:** Eva Perak, Dina Mrcela, Josko Markic

**Affiliations:** 1 Department of Emergency Medicine, University Hospital of Split, Spinciceva 1, 21000 Split, Croatia; perak.eva@gmail.com; 2 Department of Pediatrics, University Hospital of Split, Spinciceva 1, 21000 Split, Croatia; mrceladina@gmail.com; 3 School of Medicine, University of Split, Soltanska 2a, 21000 Split, Croatia

**Keywords:** diabetic ketoacidosis, COVID-19 pandemic, children, pediatric intensive care unit

## Abstract

*Background and Objectives:* Diabetic ketoacidosis (DKA) is a common complication of type 1 diabetes mellitus (T1DM) in children. Here, we explored the impact of the coronavirus disease 2019 (COVID-19) pandemic on the occurrence and severity of DKA in children in southern Croatia. *Materials and Methods:* The demographics and clinical and laboratory findings of all children and adolescents aged 0–18 years diagnosed with DKA and admitted to the pediatric intensive care unit (PICU) of the University Hospital of Split, Croatia from January 2013 to May 2023 were retrospectively collected. The participants were divided into two groups: (1) the pre-pandemic group (presenting before mid-March 2020) and (2) the pandemic group (presenting afterwards). *Results:* A total of 91 patients were included, 68 in the pre-pandemic and 23 in the pandemic group. The admission rate was similar (<1 patient per month) in both groups. In comparison to pre-pandemic patients, which mostly presented during the summer (52.9%) and winter seasons (23.5%), most pandemic cases occurred in spring (34.8%) and fall (30.4%, *p* = 0.002). No significant differences between the groups were identified in the severity of DKA, as reflected either by mean pH and median bicarbonate levels or by the proportion of patients with severe DKA. Nevertheless, HbA1c and triglycerides were significantly higher in the pandemic group (12.56% vs. 11.02%, *p* = 0.002 and 4.95 mmol/L vs. 2.8 mmol/L, *p* = 0.022, respectively) indicating poorer long-term glycemia. DKA complications were, overall, rare and without significant differences between the groups. *Conclusions:* The COVID-19 pandemic did not impact overall frequency or severity of DKA in children in southern Croatia. While the seasonal changes in DKA occurrence and a poorer long-term glycemia in pandemic patients may have been influenced by COVID-19 outbreaks and the imposed anti-pandemic measures, further studies are needed to determine if this was a temporary pandemic-related phenomenon or if this trend would persist in the future.

## 1. Introduction

Diabetic ketoacidosis (DKA) is a common complication of type 1 diabetes mellitus (T1DM) and the leading cause of mortality among diabetic children [1,2]. The reported frequency of DKA at the initial presentation of pediatric T1DM is broad and ranges from 14% to 77% of cases in some populations [3,4,5,6]. The main precipitating factors for DKA include infection, unrecognized symptoms of T1DM in previously undiagnosed cases, and, in patients with the established T1DM diagnosis, incompliance with insulin therapy largely caused by various psychosocial stressors [2,7].

In March 2020, the World Health Organization (WHO) officially announced the start of a new coronavirus disease 2019 (COVID-19) pandemic. Since then, most countries in the world introduced strict measures to combat a growing number of new cases and deaths from COVID-19. In light of this, there have been considerable changes in the organization and access to healthcare services across many countries. Stirparo et al. reported overcrowding and increased wait times in emergency medicine departments during the pandemic [8]. On the other hand, a decrease in visits and admissions for some common non-COVID-19 emergency conditions, especially in the pediatric population, was observed in some studies [9,10,11], implying that some chronic conditions might have been overlooked and undertreated. Therefore, over the past several years, there has been an increasing interest in estimating the impact of the COVID-19 pandemic on the incidence and severity of DKA in children. However, thus far, published data are controversial.

Multiple studies have reported an increased number and severity of pediatric DKA cases after the start of the pandemic [12,13,14,15,16,17,18,19,20,21]. Although the exact mechanisms to explain the latter remain unclear, a direct interference of severe acute respiratory syndrome coronavirus 2 (SARS-CoV-2) virus with the function of pancreatic β-cells [22,23], as well as indirect psychosocial effects of the pandemic measures and impaired healthcare availability [24,25], were suggested as the major contributors to the negative impact of the COVID-19 pandemic on DKA. Other studies, however, found no difference in DKA frequency pre- and post-COVID-19 [26,27,28], or found an increased DKA frequency but without difference in DKA severity [29,30,31]. Given that pandemic preventative measures and the perception of COVID-19 as a disease were different in each country, any heterogeneity or controversial findings among published studies could potentially be explained by these differences.

Several studies investigated the changes occurring in pediatric intensive care units (PICU) and found higher admission rates for DKA or severe DKA during the pandemic years [32,33,34], while a large US-based study drew a correlation between DKA admissions to PICU and periods of school closures in 2020 [35]. Interestingly, one study from the UK and Ireland found a reduction in the overall number of PICU admissions, but at the same time, a surprising 84% increase in DKA admissions during the pandemic [36].

In the Croatian pediatric population, Lah Tomulić et al. found an increase in DKA cases requiring treatment in PICU in newly diagnosed children during the first pandemic year [37]. On the other hand, a study by Vinković et al. showed no difference in either the frequency or the severity of DKA in newly diagnosed T1DM before and after the pandemic onset [38]. The aim of this study is to investigate the impact of the COVID-19 pandemic on characteristics of DKA in children hospitalized in the PICU in southern Croatia.

## 2. Materials and Methods

### 2.1. Study Design

A retrospective, single-center cross-sectional study included all consecutive patients aged 0–18 years who were admitted to the PICU of the University Hospital of Split due to DKA between 1 January 2013 and 11 May 2023. This is the only tertiary-care hospital in southern Croatia and thus serves as a critical care reference center receiving patients from the local general hospitals across the entire region. Data from forty-two patients were previously published in another study [39]. Participants were divided into two groups: the pre-pandemic group (hospitalized between 1 January 2013 and 10 March 2020, i.e., the official start of the COVID-19 pandemic), and the pandemic group (hospitalized between 11 March 2020 and 11 May 2023, i.e., the official end of the pandemic in Croatia). DKA, as an inclusion criterion, was defined according to the International Society for Pediatric and Adolescent Diabetes (ISPAD) 2022 guidelines: (1) blood glucose > 11 mmol/L, (2) venous pH < 7.3 or serum bicarbonate < 18 mmol/L, and (3) ketonemia or moderate or large ketonuria [1]. Severe DKA was defined as serum pH < 7.1 or serum bicarbonate < 5 mmol/L [1]. Patients admitted to PICU, as opposed to a regular ward, included all those with pH level < 7.2 and, additionally, those requiring intensive monitoring as per assessment of the attending pediatric emergency department physician.

The data were collected from the patients’ medical records and included information on demographic characteristics (age, sex, area of residence), season of the year at the time of admission, personal history of diabetes, family history of diabetes (including siblings, parents and grandparents), symptoms reported at admission, and vital parameters and laboratory findings at admission, as well as complications arising during the stay in the PICU. Area of residence was categorized as coastal, Dalmatian Hinterland, islands, other parts of Croatia or abroad, where the former three areas comprise the southern Croatian region. The seasons at admission included winter (from 21 December to 20 March), spring (from 21 March to 20 June), summer (from 21 June to 22 September) and fall (from 23 September to 20 December). The level of consciousness at admission was defined as normal, somnolence, sopor or coma. Blood pressure, heart rate and respiratory rate were evaluated according to the age-related reference values and were characterized as increased if equal to or above the 95th percentile [40,41]. Previous infection was recorded if the patient reported signs of infection within 15 days prior to admission. Complications were categorized as neurological, including cerebral injury; respiratory, including respiratory insufficiency; acute kidney injury; acute pancreatitis; ophthalmic complications; and death.

When calculating the frequency of DKA, to account for seasonal variations of T1DM incidence, we used the time from 11 March 2013 to 10 March 2020 as the pre-pandemic and from 11 March 2020 to 10 March 2023 as the pandemic period.

The study was approved by the Ethics Committee of the University Hospital of Split, Croatia (No. 2181-147/01-06/LJ.Z.-24-02; 22 March 2024).

### 2.2. Statistical Analysis

Statistical analysis was performed using Jeffrey’s Amazing Statistics Program (JASP) for Windows, v0.16.2 (JASP Team, Amsterdam, The Netherlands). Distribution was assessed by the Shapiro–Wilk test. The chi-squared test or Fisher’s exact test were used for categorical variables. Student’s *t*-test was used to estimate the differences between the data with normal distribution, and the Mann–Whitney U test was used for non-parametric group comparison. A *p*-value < 0.05 was considered statistically significant.

## 3. Results

A total of 91 patients were included in the study, out of which 48.4% (44/91) were males and 51.6% (47/91) were females. The median age was 10 years (IQR 7–13 years). There were 68 (74.7%) pre-pandemic and 23 (25.3%) pandemic patients. The admission rate was <1 patient per month before and during the pandemic (0.80 vs. 0.64). Patients’ characteristics are presented in Table 1. Age distribution was similar between the two subsets, except for relatively fewer children aged 0–5 years in the pandemic group. No differences regarding the region of permanent residence were observed.

The highest frequency of DKA cases before COVID-19 occurred in summer (52.9%) and winter (23.5%), while most pandemic cases occurred in spring (34.8%) and fall seasons (30.4%, *p* = 0.002). In 74.7% (68/91), the current DKA episode was the first presentation of diabetes, and this was similar in both groups (75% vs. 73.9%) (Table 1).

The distribution of most clinical symptoms, signs and vital parameters at admission was similar between the two subsets, except for less frequent vomiting (52.2% vs. 77.6%, *p* = 0.02) and more common abdominal pain (65.2% vs. 37.3%, *p* = 0.02) in the pandemic group (Table 2).

Laboratory values at admission are presented in detail in Table 3. Interestingly, the pandemic group had lower median blood glucose and ketone levels (24.7 mmol/L vs. 29.7 mmol/L, *p* = 0.022 and 5.6 mmol/L vs. 6.2 mmol/L, *p* = 0.011, respectively), as well as lower blood urea nitrogen and creatinine levels (4.3 mmol/L vs. 5.7 mmol/L, *p* = 0.044 and 46 µmol/L vs. 74 µmol/L, *p* = 0.02, respectively). Mean HbA1c and median triglyceride levels were significantly higher in the pandemic group (12.56% vs. 11.02%, *p* = 0.002 and 4.95 mmol/L vs. 2.80 mmol/L, *p* = 0.022, respectively) (Table 3).

However, there was no difference in frequency of severe DKA between the two groups, either in total or stratified by age (Table 4).

Only one patient had COVID-19 during the stay in the PICU. He had mild symptoms and recovered without COVID-related complications. Complications associated with DKA occurred in six (8.8%) patients in the pre-pandemic and one (4.3%) patient in the pandemic group (*p* = 0.674). In patients diagnosed before the pandemic, one patient had pneumomediastinum; one had acute cerebral injury, pulmonary edema, respiratory insufficiency and acute pancreatitis; two developed cerebral injury alone; and two had a nonspecific seizure attack and a transitory cataract, respectively. One patient in the pandemic group had acute cerebral injury, acute kidney injury and pulmonary edema with respiratory insufficiency. Acute cerebral injury (i.e., cerebral edema), therefore, developed in three (4.4%) pre-pandemic and in one (4.3%) pandemic patient. Only one patient, from the pre-pandemic group, died (Table 5).

## 4. Discussion

This study analyzed the changes in demographic, clinical and laboratory characteristics of children with DKA treated in the PICU that occurred after the onset of the COVID-19 pandemic, and it is the first such study originating from the southern Croatia. Children with DKA during COVID-19 presented at a similar rate and disease severity as pre-pandemic patients, even though the pandemic admissions showed a distinct seasonal distribution. Importantly, these patients had significantly higher HbA1c and triglyceride levels, indicative of a poorer long-term glycemic control. Our findings suggest that the COVID-19 pandemic has nonetheless made an impact on children with DM.

Although there were no apparent significant differences in the overall DKA frequency or patients’ demographics associated with the COVID-19 pandemic, a significant change in seasonality of DKA presentation was observed. Before the emergence of COVID-19, the highest number of patients presented during the summer months, followed by the winter season. After March 2020, however, most DKA hospitalizations occurred during spring and fall. The reasons for these changes are not entirely clear. Lavik et al. reported a higher frequency of DKA cases among both children and adults with T1DM during the first two COVID-19 waves in the United States as compared to the same periods in 2019 [42]. Considering that four out of the five reported peak COVID-19 waves in Croatia occurred predominantly the during spring and fall seasons [43,44], we conclude that the observed differences in seasonality might at least partially be attributed to the seasonal changes in pandemic outbreaks. The number of patients was, however, too small to allow for accurate monthly comparisons of DKA and COVID-19 incidences.

No significant differences in age distribution were found, although our pandemic group showed a relatively smaller proportion of very young children aged 0–5 years. This finding could be consistent with a prior report on a shift of DKA frequency towards older age groups during the pandemic [30]. However, Kamrath et al. found an increase in frequency of both DKA and severe DKA in newly diagnosed children under 6 years of age in Germany [16]. We suspect that such controversial findings could be impacted by the geographical and cultural differences pertinent to the specific daycare and school models and their ability to adapt to the pandemic. It is reasonable to suspect that the time spent at home vs. among peers would differ from region to region and therefore impact the time spent under parental supervision, as well as the likelihood of contracting COVID-19.

About three-quarters of the patients in both groups were patients with newly diagnosed DM, which is consistent with earlier studies on Croatian children [37,39]. Similarly to a study by Azova et al. [45], the ratio of newly vs. previously diagnosed patients remained the same during the pandemic, although some studies have found it to increase [30,33,46]. Children with DKA treated in the PICU more often had a negative, rather than positive, family history of DM, and this did not seem to change during the pandemic. This finding was expected, as parents with previous DM experience are usually able to recognize the symptoms sooner, before complications such as DKA occur [47,48].

Many authors suggested a delay in diagnosis of T1DM to be the main cause of worsened presentation, mainly due to hyperfocus of healthcare personnel on COVID-19 and the reluctance of parents to seek healthcare services in the midst of the pandemic crisis [24,25,27,28]. We have not observed notable changes in severity of DKA between the two groups, as reflected by similar mean pH values and median bicarbonate concentrations before and during the pandemic, as well as by similar proportions of patients with severe DKA in both groups. While our findings are consistent with some prior studies [26,29], others reported an increase in pediatric DKA severity after the start of the pandemic [15,27,33,49]. Nevertheless, despite lacking differences in severity of DKA, relatively higher HbA1c and triglyceride levels in our pandemic group indicated a poorer diabetes regulation overall, and possibly a longer duration of hyperglycemia before arriving at diagnosis in newly diagnosed patients. Indeed, worsening of long-term glycemia during the pandemic has been reported before [30,33,50]. Furthermore, a meta-analysis on pediatric DKA and T1DM, carried out by Rahmati et al., showed relatively higher mean blood glucose and HbA1c levels in children newly diagnosed with T1DM during the pandemic [12]. In a study on children and young adults with T1DM in India conducted upon the initial COVID-19 lockdown from March to May 2020, Verma et al. found significantly higher HbA1c values, and the patients reported poor compliance with insulin therapy and glucose monitoring during lockdown, mainly due to decreased availability of healthcare resources and financial problems [51]. On the contrary, several studies actually reported improvements in glycemic control in patients on continuous glucose monitoring during the first lockdown phase of the pandemic [52,53,54,55]. The authors of these studies suggested a better parental supervision during the lockdown period and the availability of telemedicine to be the main contributors to satisfactory disease management even in such unforeseen circumstances. Indeed, a large international study on patients from the SWEET registry showed that improvement or worsening of chronic glycemia in different centers correlated with the level of implementation of telemedicine, with the centers that adapted better to pandemic conditions showing better results [56].

Lastly, although nonsignificant, the overall rate of complications associated with DKA or DKA treatment was relatively lower in the pandemic group. The most common of these rare complications was acute cerebral injury (i.e., cerebral edema), known as one of the most serious complications of DKA. While the reported frequency of clinically overt cerebral injury has been low, ranging from 0.4% to 0.9% of DKA [57,58,59], it remains the cause of 60% to 90% of DKA-related deaths in children [60,61]. In our study, we found a relatively higher cerebral injury rate of 4.4%, including pre-pandemic and pandemic patients. One of those patients died, which corresponds to the previously reported fatality rates [2,62]. All four of our patients had new-onset diabetes, which is a known risk factor associated with the development of cerebral edema, most likely due to a late diagnosis [1,63]. It is, therefore, crucial to recognize diabetes symptoms early, as well as to suspect acute cerebral injury at any change in mental status.

Several studies proposed that the focus on SARS-CoV-2 during the pandemic years might have led to the under-recognition of symptoms, and therefore, the disruptions in the management of DM patients [24,25,64]. Cerovečki and Švajda reported a marked decrease in the use of DM control panels and the number of DM-related primary care visits among the overall diabetic population in Croatia in 2020 [65]. These results go along with our findings of worsened glycemic control and highlight the need to bring the general attention back to the prevention and management of common chronic diseases. One meta-analysis on 14 studies conducted worldwide reported the effectiveness of awareness-raising campaigns in decreasing the frequency of DKA at diabetes onset [66]. In the southern Croatian population, an education-based DKA prevention program conducted from April 2017 to December 2018 led to a decrease in DKA frequency among children and adolescents [67]. In these post-pandemic times, such prevention campaigns will be crucial to raise awareness of diabetes and diabetic complications, and to educate not only the patients and their parents, but also the healthcare and education facility personnel in order to enable early recognition and timely management of children with DM.

The retrospective nature of the study, the possibility of recall bias in reporting the symptoms preceding admission and a small sample size, limiting the statistical power, are some of the study’s limitations. Also, important to note is that a minor subset of patients in both groups had received initial treatment in the emergency vehicle shortly before arriving to the hospital, suggesting that differences in some of the rapidly changing laboratory values must be interpreted with caution. On the other hand, exclusion of such, typically the most severe cases, would potentially be tthe cause of an even larger bias, leading to under-recognition of clinically critical patients (such as two of our patients with cerebral injury). Lastly, we had no information on which patients were previously infected with SARS-CoV-2, either through positive PCR tests or through serologically confirmed antibodies. It is therefore impossible to draw conclusions about a direct association of SARS-CoV-2 infection and DKA.

## 5. Conclusions

Several important conclusions emerged from our analysis. We showed that there is a clear tendency towards worsened long-term glycemic control after the emergence of COVID-19, possibly related to poor disease management in children with established DM or to a longer time-to-diagnosis during the pandemic. In contrast to several prior studies, our data showed no association between the COVID-19 pandemic and the severity of DKA and no significant increase in the rate of complications, including cerebral injury. Our findings suggest that, despite indicators of worsened chronic glycemic control and possible delays in diagnosis, children still presented to the hospital in time to avoid severe deterioration of their condition. While the significant worsening of glycemic control, together with the change in seasonality of DKA, may suggest an impact of pandemic-related factors on children with DM, further larger studies, to include the post-pandemic patients, are needed to validate the latter findings. Certainly, it remains to be determined whether the observed changes were a temporary phenomenon related to the COVID-19 pandemic or if this negative trend will persist, posing a long-term problem for future disease management in diabetic children.

## Figures and Tables

**Table 1:** Demographic characteristics of the pre-pandemic vs. pandemic patients at admission.

No. (%)	Overall (*n* = 91)	Pre-Pandemic (*n* = 68)	Pandemic (*n* = 23)	*p*-Value
**Sex**				
Male	44 (48.4)	33 (48.5)	11 (47.8)	0.953
Female	47 (51.6)	35 (51.5)	12 (52.2)	
**Median age (IQR), years**	10 (7, 13)	10 (6.75, 12)	11 (9, 14)	0.126
**Age group, years**				
0–5	18 (19.8)	16 (23.5)	2 (8.7)	0.404 *
6–10	34 (37.4)	25 (36.8)	9 (39.1)	
11–15	31 (34.1)	22 (32.4)	9 (39.1)	
16–18	8 (8.8)	5 (7.4)	3 (13)	
**Area of residence**				
Coastal	47 (51.6)	35 (51.5)	12 (52.2)	0.692 *
Dalmatian Hinterland	21 (23.1)	17 (25)	4 (17.4)	
Islands	8 (8.8)	5 (7.4)	3 (13)	
Other parts of Croatia	2 (2.2)	1 (1.5)	1 (4.3)	
Abroad	13 (14.3)	10 (14.7)	3 (13)	
**Season of the year**				
Winter	20 (22)	16 (23.5)	4 (17.4)	0.002 *
Spring	15 (16.5)	7 (10.3)	8 (34.8)	
Summer	40 (44)	36 (52.9)	4 (17.4)	
Fall	16 (17.6)	9 (13.2)	7 (30.4)	
**Diabetes status**				
Newly diagnosed	68 (74.7)	51 (75)	17 (73.9)	0.917
Previously diagnosed	23 (25.3)	17 (25)	6 (26.1)	
**Family history of diabetes ^†^**				
Negative	49 (55.7)	36 (55.4)	13 (56.5)	0.925
Positive	39 (44.3)	29 (44.6)	10 (43.5)	

* *p*-value was calculated using Fisher’s exact test. ^†^ Overall number of patients with available data was 88 (65 pre-pandemic and 23 pandemic).

**Table 2:** Clinical signs, symptoms and vital parameters of the pre-pandemic vs. pandemic patients at admission.

Symptom/Sign	Total No. of Patients ^†^			No. with Symptom/Sign (%)			*p*-Value
	Overall	Pre-Pandemic	Pandemic	Overall	Pre-Pandemic	Pandemic	
Polydipsia	90	67	23	57 (63.3)	42 (62.7)	15 (65.2)	0.828
Polyuria	90	67	23	50 (55.6)	38 (56.7)	12 (52.2)	0.705
Polyphagia	90	67	23	13 (14.4)	7 (10.4)	6 (26.1)	0.066
Nocturia	89	66	23	37 (41.6)	26 (39.4)	11 (47.8)	0.480
Nausea	90	67	23	11 (12.2)	6 (9)	5 (21.7)	0.106
Vomiting	90	67	23	64 (71.1)	52 (77.6)	12 (52.2)	0.02
Abdominal pain	90	67	23	40 (44.4)	25 (37.3)	15 (65.2)	0.02
Diarrhea	90	67	23	8 (8.9)	8 (11.9)	0 (0)	0.108 *
Fatigue	90	67	23	45 (50)	33 (49.3)	12 (52.2)	0.809
Headache	90	67	23	5 (5.6)	3 (4.5)	2 (8.7)	0.599 *
Weight loss	86	63	23	47 (54.7)	34 (54)	13 (56.5)	0.833
Kussmaul breathing	87	64	23	51 (58.6)	36 (56.3)	15 (65.2)	0.454
Acetone breath	90	67	23	36 (40)	28 (41.8)	8 (34.8)	0.554
Dehydration	89	66	23	81 (91)	59 (89.4)	22 (95.7)	0.675 *
Paleness	90	67	23	46 (51.1)	35 (52.2)	11 (47.8)	0.715
Axillary temperature							
Afebrile	84	61	23	68 (81)	49 (80.3)	19 (82.6)	1 *
Subfebrile/febrile				16 (19)	12 (19.7)	4 (17.4)	
Tachycardia	90	67	23	41 (45.6)	32 (47.8)	9 (39.1)	0.473
Tachypnea	69	48	21	34 (49.3)	25 (52.1)	9 (42.9)	0.481
Hypertension	73	52	21	37 (50.7)	23 (44.2)	14 (66.7)	0.083
Disorder of consciousness:							
Yes	90	68	22	39 (43.3)	29 (42.6)	10 (45.5)	0.817
No				51 (56.7)	39 (57.4)	12 (54.5)	
Somnolence				32 (35.6)	25 (36.8)	7 (31.8)	0.487 *
Sopor				6 (6.7)	3 (4.4)	3 (13.6)	
Coma				1 (1.1)	1 (1.5)	0 (0)	
Previous infection	90	67	23	36 (40)	26 (38.8)	10 (43.5)	0.693

^†^ Number of patients in each group for whom data were available. * *p*-value was calculated using Fisher’s exact test.

**Table 3:** Laboratory values of the pre-pandemic vs. pandemic patients at admission.

Laboratory Parameter	Total No. of Patients ^†^			Median (IQR) Laboratory Value			*p*-Value
	Overall	Pre-Pandemic	Pandemic	Overall	Pre-Pandemic	Pandemic	
**Blood glucose, mmol/L**	90	67	23	28.35 (23.55, 35.4)	29.7 (23.7, 38.9)	24.7 (23.1, 28.8)	0.022
**Blood ketones, mmol/L**	72	55	17	5.95 (5.6, 6.73)	6.2 (5.65, 6.9)	5.6 (5.1, 6.1)	0.011
HbA1c, % *	77	55	22	11.46 ± 2.03	11.02 ± 1.85	12.56 ± 2.1	0.002
pH *	91	68	23	7.09 ± 0.12	7.09 ± 0.12	7.09 ± 0.12	0.779
**pCO_2_, kPa**	86	63	23	2.02 (1.6, 2.77)	2 (1.6, 2.74)	2.05 (1.65, 2.68)	0.992
**pO_2_, kPa**	82	59	23	10.7 (8.45, 12.45)	11.2 (8.85, 12.75)	9.6 (8.32, 10.95)	0.058
**HCO_3_, mmol/L**	90	68	22	4.4 (3.1, 7)	4.35 (3.1, 7.2)	4.75 (3.15, 6.28)	0.940
**BE, mmol/L**	82	62	20	−23.3 (−24.88, −19.93)	−23.4 (−9.8, −19.6)	−23.2 (−24.4, −21.08)	0.966
**Anion gap, mmol/L**	88	67	21	26.8 (22.43, 29.6)	26.5 (21.9, 29.6)	29 (25.2, 32.7)	0.082
**Na, mmol/L**	89	67	22	134 (131, 137)	134 (130, 136)	135 (134, 137)	0.025
**K, mmol/L**	90	67	23	4.5 (4, 5)	4.6 (4.1, 5.05)	4.3 (3.85, 4.75)	0.071
**Cl, mmol/L**	90	67	23	102 (97, 105)	102 (96, 105)	101 (99, 104)	0.93
Ca, mmol/L *	87	65	22	2.44 ± 0.18	2.45 ± 0.18	2.42 ± 0.19	0.398
**P, mmol/L**	86	64	22	1.25 (1.077, 1.58)	1.31 (1.07, 1.57)	1.19 (1.05, 1.42)	0.342
**BUN, mmol/L**	87	64	23	5.4 (4.3, 7.2)	5.7 (4.68, 7.58)	4.3 (3.4, 6.8)	0.044
**Creatinine, µmol/L**	87	64	23	69 (46.5, 98)	74 (54, 99.25)	46 (38, 85)	0.02
**Leukocytes, ×10^9^/L**	89	66	23	17.5 (11.4, 24.2)	17.4 (13.13, 24.43)	17.6 (10.57, 23.5)	0.866
Cholesterol, mmol/L *	74	52	22	5.37 ± 1.54	5.16 ± 1.34	5.85 ± 1.88	0.081
**Triglycerides, mmol/L**	74	52	22	3.65 (2.2, 5.58)	2.8 (2.18, 5.13)	4.95 (2.65, 8.38)	0.022
**HDL, mmol/L**	73	52	21	1 (0.8, 1.3)	0.9 (0.8, 1.3)	1 (0.8, 1.2)	0.633
**LDL, mmol/L**	73	52	21	2.6 (1.9, 3.1)	2.68 (1.98, 3.2)	2.5 (1.8, 2.97)	0.374

^†^ Number of patients in each group for whom data were available. * Values were calculated using Student’s *t*-test and are presented as mean ± standard deviation. Abbreviations: IQR—interquartile range; HbA1c—glycated hemoglobin; pCO_2_—partial pressure of carbon dioxide; pO_2_—partial pressure of oxygen; HCO_3_—bicarbonate; BE—base excess; Na—sodium; K—potassium; Cl—chloride; Ca—calcium; P—phosphorus; BUN—blood urea nitrogen; HDL—high-density lipoprotein; LDL—low-density lipoprotein.

**Table 4:** DKA severity in the pre-pandemic vs. pandemic patients.

No. (%)	Overall (*n* = 91)	Pre-Pandemic (*n* = 68)	Pandemic (*n* = 23)	*p*-Value
Severe DKA	58 (63.7)	42 (61.8)	16 (69.6)	0.501
Severe DKA by age groups, years				
0–5	12 (20.7)	10 (23.8)	2 (12.5)	0.386 *
6–10	22 (37.9)	16 (38.1)	6 (37.5)	
11–15	19 (32.8)	14 (33.3)	5 (31.3)	
16–18	5 (8.6)	2 (4.8)	3 (18.8)	

* *p*-value was calculated using Fisher’s exact test.

**Table 5:** DKA complication rates in the pre-pandemic vs. pandemic patients.

No. (%)	Overall (*n* = 91)	Pre-Pandemic (*n* = 68)	Pandemic (*n* = 23)	*p*-Value
Complications of DKA				
Yes	7 (7.7)	6 (8.8) ^†^	1 (4.3) ^†^	0.674 *
No	84 (92.3)	62 (91.2)	22 (95.7)	
Cerebral injury	4 (4.4)	3 (4.4)	1 (4.3)	-
Acute kidney injury	1 (1.1)	0 (0)	1 (4.3)	-
Pneumomediastinum	1 (1.1)	1 (1.5)	0 (0)	-
Acute pancreatitis	1 (1.1)	1 (1.5)	0 (0)	-
Pulmonary edema	2 (2.2)	1 (1.5)	1 (4.3)	-
Acute respiratory insufficiency	2 (2.2)	1 (1.5)	1 (4.3)	-
Nonspecific seizure attack	1 (1.1)	1 (1.5)	0 (0)	-
Transitory cataract	1 (1.1)	1 (1.5)	0 (0)	-
Death	1 (1.1)	1 (1.5)	0 (0)	-

^†^ Several patients displayed more than one complication. Therefore, the total number of patients with any complication is smaller than the sum of individual complication’s frequencies. * *p*-value was calculated using Fisher’s exact test.

## Data Availability

The data presented in this study are available on request from the corresponding author.
